# Altered Monocyte Populations and Activation Marker Expression in Children with Autism and Co-Occurring Gastrointestinal Symptoms

**DOI:** 10.3390/biom15020207

**Published:** 2025-02-01

**Authors:** Rachel J. Moreno, Yasmin W. Azzam, Serena Eng, Destanie Rose, Paul Ashwood

**Affiliations:** 1 Department of Medical Microbiology and Immunology, University of California, Davis, CA 95616, USA; 2 MIND Institute, University of California, Sacramento, CA 95817, USA

**Keywords:** autism, monocytes, innate immunity, gastrointestinal symptoms, GI, gut, gut-brain-axis, myeloid cells, monocyte subpopulations, behavior, neurodevelopment, inflammation, immune

## Abstract

Autism spectrum disorder (ASD) is an early-onset neurodevelopmental condition that now impacts 1 in 36 children in the United States and is characterized by deficits in social communication, repetitive behaviors, and restricted interests. Children with ASD also frequently experience co-morbidities including anxiety and ADHD, and up to 80% experience gastrointestinal (GI) symptoms such as constipation, diarrhea, and/or abdominal pain. Systemic immune activation and dysregulation, including increased pro-inflammatory cytokines, are frequently observed in ASD. Evidence has shown that the innate immune system may be impacted in ASD, as altered monocyte gene expression profiles and cytokine responses to pattern recognition ligands have been observed compared to typically developing (TD) children. In humans, circulating monocytes are often categorized into three subpopulations—classical, transitional (or “intermediate”), and nonclassical monocytes, which can vary in functions, including archetypal inflammatory and/or reparative functions, as well as their effector locations. The potential for monocytes to contribute to immune dysregulation in ASD and its comorbidities has so far not been extensively studied. This study aims to determine whether these monocyte subsets differ in frequency in children with ASD and if the presence of GI symptoms alters subset distribution, as has been seen for T cell subsets. Whole blood from ASD children with (ASD^+^GI^+^) and without gastrointestinal symptoms (ASD^+^GI^−^) and their TD counterparts was collected from children enrolled in the Childhood Autism Risk from Genetics and Environment (CHARGE) study. Peripheral blood mononuclear cells were isolated and stained for commonly used subset identifiers CD14 and CD16 as well as activation state markers CCR2, HLA-DR, PD-1, and PD-L1 for flow cytometry analysis. We identified changes in monocyte subpopulations and their expression of surface markers in children with ASD compared to TD children. These differences in ASD appear to be dependent on the presence or absence of GI symptoms. We found that the ASD^+^GI^+^ group have a different monocyte composition, evident in their classical, transitional, and nonclassical populations, compared to the ASD^+^GI^−^ and TD groups. Both the ASD^+^GI^+^ and ASD^+^GI^−^ groups exhibited greater frequencies of classical monocytes compared to the TD group. However, the ASD^+^GI^+^ group demonstrated lower frequencies of transitional and nonclassical monocytes than their ASD^+^GI^−^ and TD counterparts. CCR2^+^ classical monocyte frequencies were highest in the ASD^+^GI^−^ group. HLA-DR^+^ classical, transitional, and nonclassical monocytes were statistically comparable between groups, however, HLA-DR^−^ nonclassical monocyte frequencies were lower in both ASD groups compared to TD. The frequency of classical monocytes displaying exhaustion markers PD-1 and PD-L1 were increased in the ASD^+^GI^+^ group compared to ASD^+^GI^−^ and TD, suggesting potentially impaired ability for clearance of foreign pathogens or debris, typically associated with worsened inflammation. Taken together, the findings of differential proportions of the monocyte subpopulations and altered surface markers may explain some of the characteristics of immune dysregulation, such as in the gastrointestinal tract, observed in ASD.

## 1. Introduction

Recent data from the Centers for Disease Control and Prevention (CDC) estimates 1 in 36 children are diagnosed with autism spectrum disorder (ASD) [1]. A clinical diagnosis of ASD is made when social and communicative deficiencies are present along with restrictive and repetitive behaviors. ASD is an early-onset and heterogeneous neurodevelopmental condition that impacts the individual’s ability to learn, communicate, relate to others, and maintain social interactions [2,3]. Mounting evidence over the last 40 years has suggested that there is immune dysregulation in ASD. Increases in inflammatory mediators in the peripheral blood and central nervous system (CNS), genetic mutations, and differential gene expression in genes associated with the immune response are often present in ASD [4,5,6]. For instance, the Akt/mammalian target of rapamycin (mTOR) pathway—which is integral to many cellular processes, including cell growth, immune function, and neurodevelopment—has been demonstrated to be more highly active in cells from children with ASD [7]. Dysregulation of the mTOR pathway has been suggested by multiple studies to contribute to the development of ASD due to its involvement in neuronal migration, axon formation, synaptic activity, and plasticity [8]. Furthermore, increased immune dysregulation in children with ASD is associated with worsened behavioral impairments [9,10]. For example, levels of inflammatory cytokines IL-1β and IL-6 were elevated in ASD children and associated with worsened irritability according to the Aberrant Behavior Checklist (ABC) and Autism Diagnostic Observation Schedule (ADOS) assessments [10,11,12].

Children with ASD are far more likely than typically developing (TD) children to experience comorbidities, including gastrointestinal (GI) symptoms such as constipation, diarrhea, and abdominal pain [9]. In addition, ASD children with GI symptoms (ASD^+^GI^+^) have different inflammatory cytokine profiles and T cell phenotypes than ASD children without GI symptoms (ASD^+^GI^−^) [9,13,14]. In ASD^+^GI^+^ children, increased intestinal inflammation has been observed, such as increased inflammatory cell infiltration into the gut tissue and elevated frequencies of mucosal CD3^+^IL-6^+^ and CD3^+^TNFα^+^ cells, relative to TD controls with similar GI symptoms [15,16]. Moreover, peripheral blood mononuclear cells (PBMC) isolated from children with ASD^+^GI^+^ and subsequently stimulated with Toll-like receptor (TLR)-ligands or mitogens produced increased levels of mucosa-related inflammatory cytokines IL-5, IL-15, and IL-17 and decreased levels of regulatory cytokine TGF-β1 in comparison to their ASD^+^GI^−^ and TD counterparts [13]. In ASD^+^GI^+^ children, increased frequencies of T helper 17 cells (T_H_17) and decreases in gut-homing regulatory T cells (T_regs_) were also observed relative to ASD^+^GI^−^ and TD groups [9]. Taken together, these data indicate that inflammatory dysregulation may be exacerbated in children with ASD and co-occurring GI symptoms. While the adaptive immune system has been studied in the context of GI issues in ASD, innate immune dysregulation has not been as extensively investigated [17,18]. Therefore, the role of innate immune cells in GI-related immune dysregulation of ASD children remains to be understood.

Monocytes are a heterogeneous population of immature mononuclear phagocytes that comprise 5–10% of circulating cells that can be recruited to multiple tissue sites through finely orchestrated extracellular and intracellular signals that cue reparative or inflammatory phenotypes [19]. As circulating phagocytes, they exhibit a high degree of functional dynamism that normally permits the detection of endogenous and exogenous danger and pathogen-associated molecular patterns (i.e., lipid particles, microbes, and apoptotic and necrotic cell debris) to maintain multi-organ tissue homeostasis. However, chronic stimulation and persistent activation of circulating monocytes propagate and progress many debilitating inflammatory conditions [20]. Monocytes have been shown to be dysfunctional in ASD, including the increased production of inflammatory cytokines after stimulation with TLR ligands relative to TD [21,22]. Monocytes from children with ASD responded differently to immunogens and have issues in regulating immune activation as identified by transcriptional studies [21,22,23]. Furthermore, RNA sequencing studies of stimulated monocytes from children with ASD showed enriched expression of genes associated with activation of pathways involving immune activation and chemotaxis [22]. Interestingly, this was accompanied by a lack of downregulation of genes involved in protein synthesis and transcriptional machinery, suggesting impaired resolution of immune response in the ASD group [22].

Monocytes have previously been categorized into three subpopulations—classical, transitional, and nonclassical monocytes—that differ in cell surface marker distribution, frequency in the blood, and effector functions [20]. Human monocyte subsets are commonly defined by their surface expression of CD14 and CD16, with CD14 expression highest on classical monocytes and largely diminished on nonclassical monocytes, while the inverse is true for CD16 [20,24]. Transitional monocytes have an intermediate expression of both markers [20,24]. Thus, classical (CD14^+^CD16^−^), transitional (CD14^+^CD16^+^), and nonclassical (CD14^lo^CD16^+^) monocytes are often referred to by this distribution of markers, as is described henceforth in this paper. The expression of the C-C chemokine receptor type 2 (CCR2), human leukocyte antigen-DR (HLA-DR), and more recently the immunosuppressive immune checkpoint markers programmed death-1 (PD-1) and programmed death-ligand 1 (PD-L1) can aid in further defining these monocyte populations more precisely and identifying their levels of activation [25]. CCR2 is known to facilitate the extravasation, adhesion, and migration of monocytes to sites of inflammation, including the brain in CNS diseases such as multiple sclerosis and Alzheimer’s disease [26]. CCR2 is most highly expressed on classical monocytes, at medium levels on transitional monocytes, and at very low levels on nonclassical monocytes [1]. HLA-DR, an MHC class II cell surface receptor, is a crucial molecule for antigen presentation and a marker of the immune competency of the host. HLA-DR has been demonstrated to be constitutively expressed on CD16^+^ monocytes (i.e., transitional and nonclassical) and may be diminished in classical monocytes [27]. Expression of molecules PD-1 and PD-L1 on immune cells has been linked with cellular exhaustion, apoptosis, and higher prognosis of a variety of immune diseases as well as neuroinflammation [28,29]. The upregulation of these markers on monocytes has been associated with impaired ability for clearance of foreign pathogens and associated with worsened inflammation [30]. For example, PD-1 upregulation on monocytes in the blood has been observed after acute ischemic stroke and shows potential as a therapeutic target for neuroinflammatory diseases [31].

A number of studies have collectively shown that children with ASD have higher frequencies of monocytes, although the heterogeneity within the monocyte subpopulations has not been investigated in the context of ASD [32]. Furthermore, the heterogeneity in monocyte subpopulations and their different roles in homeostasis has the potential to shed light on the relationship between GI comorbidities in ASD individuals. This study aims to provide insight into how monocyte populations and their marker expression are changed in ASD when GI symptoms are present.

## 2. Material and Methods

### 2.1. Study Participants

Thirty-four children ages 4 to 9 years old previously enrolled in the Childhood Autism Risk from Genetics and Environment (CHARGE) study were consecutively recruited as the study population (Table 1). Experimental groups were defined as ASD children with GI symptoms (ASD^+^GI^+^), ASD children with no GI symptoms (ASD^+^GI^−^), and typically developing (TD) children without GI symptoms. As described in prior publications, an ASD diagnosis was confirmed by trained behavioral specialists at the UC Davis MIND Institute using the Autism Diagnostic Interview-Revised (ADI-R) and the Autism Diagnostic Observation Schedule (ADOS). TD children were screened and included after assessment with the Social Communication Questionnaire (excluded if scores > 11) (SCQ−Lifetime Edition) ruled out ASD risk. Further behavioral characteristics for the TD and ASD groups were obtained using the Aberrant Behavior Checklist (ABC) to measure irritability, lethargy, stereotypic behaviors, and inappropriate speech (Table 1). Exclusion criteria for subjects included a previous diagnosis of a GI disease (celiac disease or inflammatory bowel disease), genetic disorders, liver or pancreatic disease, cystic fibrosis, infections, and/or current fever. Children receiving dietary interventions under the supervision of a dietician/nutritionist were also excluded, except children who had nutritional modifications not under the supervision of a healthcare professional.

### 2.2. GI Symptom Evaluation

GI evaluations used for this study are discussed extensively in prior publications [13]. In brief, GI symptoms for all groups were evaluated using the CHARGE GI History (GIH) survey and GI symptom survey (GISS) based on the Rome III Diagnostic Questionnaire for the Pediatric Functional GI Disorders. The GIH measure ten types of GI symptoms, consisting of abdominal pain, gaseousness or bloating, sensitivity, diarrhea, constipation, pain on stooling, vomiting, sensitivity to foods, difficulty swallowing, blood in stools, and blood in vomit, which are scored on the Likert scale [(0) = never, (1) = rarely, (2) = sometimes, (3) = frequently and (4) = always]. The GISS survey consists of 7 sections that evaluate the research subject’s constipation, diarrhea, or irritable bowel syndrome (IBS). Research participants identified as having irregular bowel habits of constipation, diarrhea, or IBS on the GI surveys were placed in the ASD group with GI issues (ASD^+^GI^+^), while those that did not were placed in the ASD group with no GI group (ASD^+^GI^−^).

This study was approved by the institutional review boards for the State of California and the University of California, Davis. Written and informed consents were obtained from research participants legal guardians prior to blood draw and data collection in accordance with the UC Davis IRB protocol.

### 2.3. Blood Collection and Flow Cytometry

Peripheral blood was collected in acid-citrate dextrose Vacutainers (BD Biosciences, San Jose, CA, USA). Blood was spun at 2100 rpm for 10 min, followed by plasma removal. PBMC were subsequently isolated by layering the remaining blood components onto lymphocyte separation medium (StemCell) and spinning at 1700 rpm for 30 min, followed by the removal of the buffy coat. PBMC were subsequently washed twice with Hanks Balanced Salt Solution (HBSS). PBMC (1 × 10^6^) were removed and used for flow cytometry. PBMC were resuspended in 100 µL of live/dead amine dye (live/dead Fixable Aqua Dead Cell Strain Kit, 405 nm excitation; Invitrogen) for 15 min. Cells were washed twice with PBMC wash (PBS, sodium azide, and BSA). 100 µL of Fc Block (1:200 dilution) and 5 µL of True-Stain Monocyte blocker were added and incubated for 5 min, followed by the incubation with anti-CD14 (BV421), anti-CD192 (CCR2) (AF488), anti-HLA-DR (APC/Fire 750), anti-CD16 (APC), anti-CD279 (PD-1) (PE), and anti-CD274 (PD-L1) (PerCP-Cy5.5) for 25 min (Table 2). PBMC were washed twice with PBMC wash and resuspended in 2% PFA (Sigma-Aldrich, St. Louis, MO, USA) for 30 min. After incubation, cells were washed with PBMC wash, followed by an FACS fluid wash and resuspension in 200 µL of FACS fluid. Flow cytometric acquisition was performed on an LSR II flow cytometer (BD Biosciences) using FACSDiva software version 6.0 (BD Biosciences) with 100,000 events collected for each sample. Event analysis was done using FlowJo software version 9.0, and monocytes were identified using forward and side scatter parameters, with debris removed using singlet and live/dead gates.

### 2.4. Statistics

Statistical analysis was performed using GraphPad Prism (Version 10.1.0). ROUT outlier removal (Q = 1%) was applied to all data. Ordinary one-way ANOVA was used on data to determine statistical significance in the monocyte subpopulation frequencies across experimental groups. Tukey’s multiple comparisons test was used to correct for multiple comparisons, ensuring that all reported *p*-values are adjusted. Statistical significance was noted in the figures as *p*-values < 0.05 and error bars representing SEM.

## 3. Results

### 3.1. Children with ASD with GI Comorbidities Have Altered Frequencies of Monocyte Subpopulations

**Figure 1:**
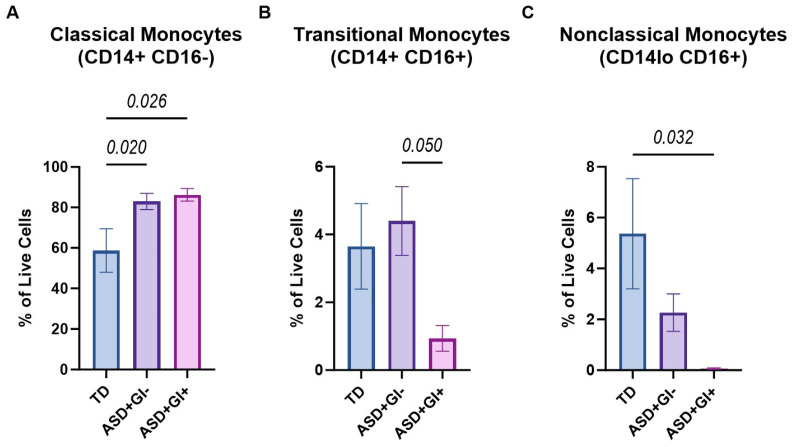
Monocyte subpopulation frequencies based on diagnosis and GI status. The percentage of live cells from each monocyte subpopulation—(**A**) classical (CD14^+^CD16^−^), (**B**) transitional (CD14^+^CD16^+^), and (**C**) nonclassical (CD14^lo^CD16^+^)—was identified using flow cytometry based on CD14 and CD16 expression in TD, ASD^+^GI^−^, and ASD^+^GI^+^ groups. ROUT outlier removal (Q = 1%) was applied, and statistical significance between groups (*p* < 0.05) was determined using ordinary one-way ANOVA and Tukey’s multiple comparisons test. Error bars represent SEM.

The frequencies of live classical (CD14^+^CD16^−^), transitional (CD14^+^CD16^+^), and nonclassical (CD14^lo^CD16^+^) cells from all samples were compared between TD, ASD^+^GI^−^, and ASD^+^GI^+^ groups for all three monocyte subpopulations. Both the ASD^+^GI^−^ (Mean ± SEM; 82.96 ± 3.98%, *p* = 0.020) and ASD^+^GI^+^ (86.23 ± 3.13%, *p* = 0.026) groups had significantly higher frequencies of classical monocytes compared to the TD group (58.70 ± 10.78%) with no statistically significant difference noted between the ASD^+^ groups (Figure 1A). Conversely, the ASD^+^GI^+^ group (0.93 ± 0.38%) had lower frequencies of transitional monocytes compared to the ASD^+^GI^−^ (4.40 ± 1.02%, *p* = 0.050) group and were also decreased compared to the TD (3.65 ± 1.26%, *p* = 0.172) group, although the latter comparison did not reach statistical significance (Figure 1B). The ASD^+^GI^+^ group (0.06 ± 0.03%) had lower frequencies of nonclassical monocytes compared to the TD (5.37 ± 2.17%, *p* = 0.032) and trended towards lower frequencies compared to the ASD^+^GI^−^ (2.27 ± 0.74%) group but did not reach statistical significance (Figure 1C). There were no significant differences in either transitional or nonclassical monocyte frequencies between the TD and ASD^+^GI^−^ groups.

### 3.2. The Frequency of Monocytes with the Inflammatory Marker CCR2 Was Elevated in Children with ASD and Altered Based on GI Status

**Figure 2:**
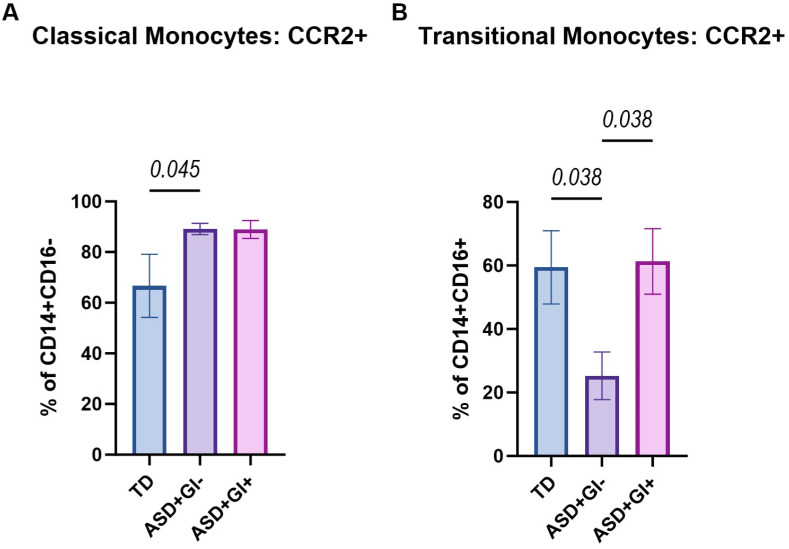
CCR2 expression on monocyte subpopulations based on diagnosis and GI status. The percentage of CCR2-expressing cells from (**A**) classical (CD14^+^CD16^−^) and (**B**) transitional (CD14^+^CD16^+^) monocyte subpopulations was identified using flow cytometry in TD, ASD^+^GI^−^, and ASD^+^GI^+^ groups. ROUT outlier removal (Q = 1%) was applied, and statistical significance between groups (*p* < 0.05) was determined using ordinary one-way ANOVA and Tukey’s multiple comparisons test. Nonclassical (CD14^lo^CD16^+^) monocytes were excluded from the figure due to an insufficient number of captured events in the ASD^+^GI^+^ group required for statistical analysis. Error bars represent SEM.

The frequencies of monocytes with cell surface CCR2^+^ were assessed next. CCR2^+^ on classical (CD14^+^CD16^−^CCR2^hi^) and transitional (CD14^+^CD16^+^CCR2^hi^) monocytes were compared between TD, ASD^+^GI^−^, and ASD^+^GI^+^ groups. As is biologically expected due to low numbers of nonclassical monocytes in the ASD^+^GI^+^ group, nonclassical monocytes expressing CCR2 (CD14^lo^CD16^+^CCR2^hi^) were excluded due to an insufficient number of captured events required for statistical analysis. The frequency of CCR2^+^ classical monocyte levels was significantly higher in the ASD^+^GI^−^ group (89.11 ± 2.26%) compared to the TD group (66.66 ± 12.44%, *p* = 0.045) (Figure 2A). No differences were seen between ASD^+^GI^+^ (88.92 ± 3.57%) and ASD^+^GI^−^ groups. Although CCR2^+^ on classical monocyte was increased in the ASD^+^GI^+^ group compared to the TD groups, this did not meet statistical significance (*p* = 0.136), with the TD group exhibiting more variation in the frequency of CCR2^+^ cells than the ASD^+^GI^+^ group. Conversely, the frequency of CCR2^+^ transitional monocyte levels was lower in the ASD^+^GI^−^ (25.24 ± 7.50%) group compared to both the TD (59.43 ± 11.55%, *p* = 0.038) and ASD^+^GI^+^ (61.30 ± 10.32%, *p* = 0.038) groups (Figure 2B). The frequencies of CCR2^+^ transitional monocytes were comparable between the TD and ASD^+^GI^+^ groups.

### 3.3. Frequencies of Monocytes with Activation Markers of HLA-DR, PD-1, and PD-L1 Differed Between Children with ASD

**Figure 3:**
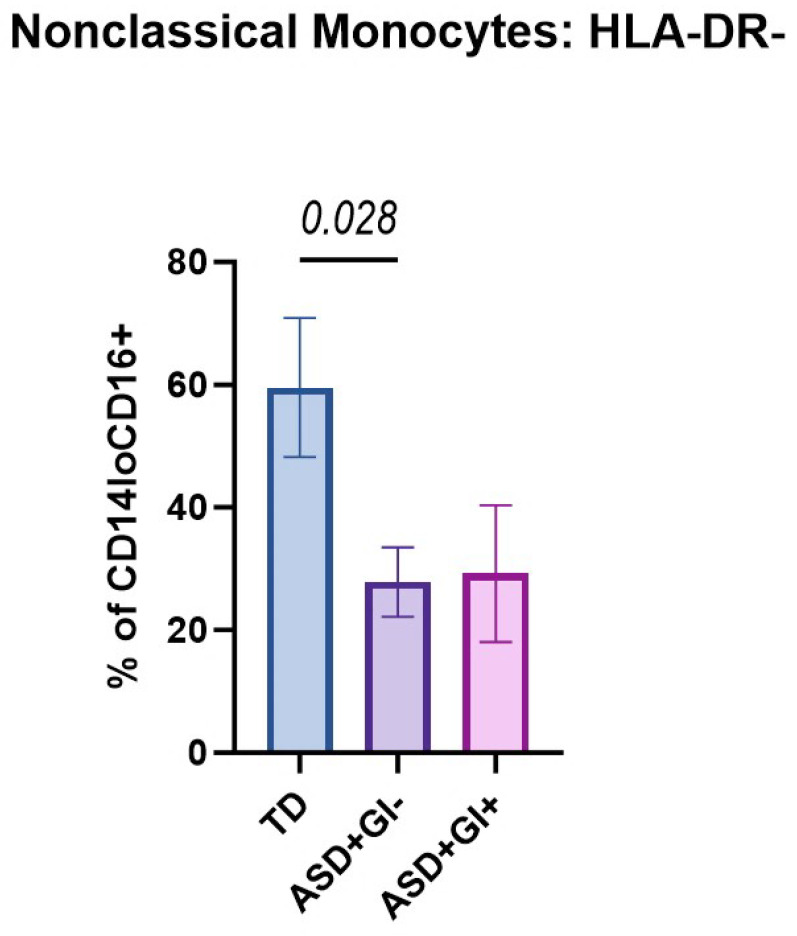
HLA-DR expression on nonclassical monocyte populations based on ASD diagnosis and GI status. The percentage of HLA-DR non-expressing nonclassical (CD14^lo^CD16^+^) monocytes was identified using flow cytometry in the TD, ASD^+^GI^−^, and ASD^+^GI^+^ groups. HLA-DR^+^ classical (CD14^+^CD16^−^), transitional (CD14^+^CD16^+^), and nonclassical cells, as well as HLA-DR^−^ classical and transitional cells, did not significantly differ across the three groups. ROUT outlier removal (Q = 1%) was applied, and statistical significance between groups (*p* < 0.05) was determined using ordinary one-way ANOVA and Tukey’s multiple comparisons test. Error bars represent SEM.

To assess the frequency of monocytes with differential patterns of activation markers, we evaluated HLA-DR, PD-1, and PD-L1. The frequencies of HLA-DR^+^ and HLA-DR^−^ classical, transitional, and nonclassical monocytes were compared between TD, ASD^+^GI^−^, and ASD^+^GI^+^ groups. No significant differences in frequencies of HLA-DR^+^ classical, transitional, and nonclassical monocytes or HLA-DR^−^ classical and transitional monocytes were observed across groups (Table 3). However, differences in the frequencies of nonclassical HLA-DR^−^ monocytes (CD14^lo^CD16^+^ HLA-DR^lo^) were observed between the groups (Figure 3). The ASD^+^GI^−^ group (27.82 ± 5.66%) had lower frequencies of HLA-DR^−^ nonclassical monocytes compared to the TD group (59.58 ± 11.32%, *p* = 0.028). ASD^+^GI^−^ and ASD^+^GI^+^ (29.20 ± 11.18%) groups had comparable frequencies of HLA-DR^−^ nonclassical monocytes. A trend was observed for a lower frequency of HLA-DR^−^ nonclassical monocyte frequencies between the ASD^+^GI^+^ and TD groups (*p* = 0.086).

The frequencies of PD-1^+^, PD-L1^+^, and PD-1^+^PD-L1^+^ classical and transitional monocytes were compared between TD, ASD^+^GI^−^, and ASD^+^GI^+^ groups.

**Figure 4:**
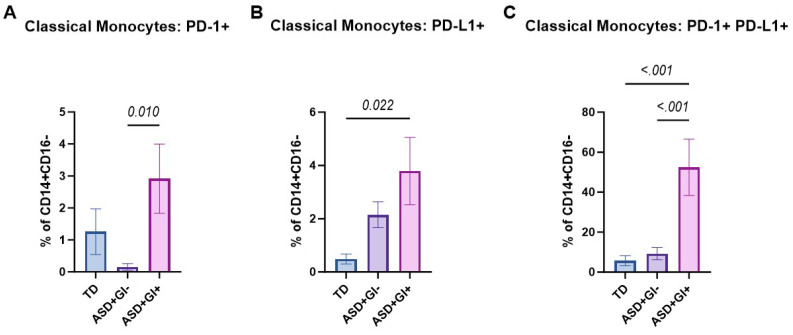
PD-1, PD-L1, and PD-1 PD-L1 co-expression on classical monocyte populations based on ASD diagnosis and GI status. The percentage of (**A**) PD-1, (**B**) PD-L1, and (**C**) PD-1 PD-L1 co-expressing cells from classical (CD14^+^CD16^−^) monocyte populations were identified using flow cytometry in TD, ASD^+^GI^−^, and ASD^+^GI^+^ groups. ROUT outlier removal (Q = 1%) was applied, and statistical significance between groups (*p* < 0.05) was determined using ordinary one-way ANOVA and Tukey’s multiple comparisons test. PD-1, PD-L1, and PD-1 PD-L1 co-expressing transitional (CD14^+^CD16^+^) monocytes did not significantly differ across the three groups. Nonclassical (CD14^lo^CD16^+^) monocytes were excluded from the figure due to an insufficient number of captured events in the ASD^+^GI^+^ group required for statistical analysis. Error bars represent SEM.

No significant differences in frequencies of PD-1, PD-L1, and PD-1 PD-L1 co-expressing transitional monocytes were observed across groups (Table 3). Nonclassical monocytes were excluded from analysis due to an insufficient number of captured events. PD-1^+^ classical monocyte frequencies were comparable between the TD (1.26 ± 0.72%) and ASD^+^GI^−^ (0.16 ± 0.11%) groups (Figure 4A). However, PD-1^+^ classical monocytes were increased in the ASD^+^GI^+^ (2.92 ± 1.08%) group compared to the ASD^+^GI^−^ group (*p* = 0.010). PD-L1^+^ classical monocyte levels were comparable between the TD (0.49 ± 0.19%) and ASD^+^GI^−^ (2.15 ± 0.49%) groups (Figure 4B). However, the ASD^+^GI^+^ (3.79 ± 1.26%) group had markedly higher PD-L1^+^ classical monocyte levels than the TD group (*p* = 0.020). When assessing PD-1 and PD-L1 double positive cells, once again classical monocyte levels were comparable between the TD (5.73 ± 2.50%) and ASD^+^GI^−^ (9.26 ± 3.06%) groups. However, the ASD^+^GI^+^ (52.43 ± 14.08%) group had markedly elevated frequencies of PD-1^+^PD-L1^+^ classical monocytes than both the TD and ASD^+^GI^−^ groups (*p* < 0.001) (Figure 4C).

## 4. Discussion

Immune system dysfunction occurring in individuals with ASD could explain why a substantial number experience immune-mediated co-occurring conditions such as GI issues, food allergies, inflammatory bowel disease, autoimmunity, and asthma. Evidence of increased innate immune cytokines such as IL-1β, TNFα, and monocyte chemoattractant protein-1 (MCP-1) in the periphery of children with ASD suggests that dysfunctional innate immune cells could be responsible [6,33]. Monocytes isolated from children with ASD have differential cytokine responses when stimulated with immunogens, implicating them as a potential contributor to, if not the source of, inflammatory markers seen in the circulating blood [21]. Monocytes are classified into three subsets with overlapping as well as distinct functions: classical, transitional, and nonclassical. For the first time, we have identified changes in all three of these monocyte subpopulations and their expression of surface markers in children with ASD compared to TD children. These differences in ASD appear to be dependent on the presence or absence of GI symptoms. More specifically, children with ASD and comorbid GI issues were found to have increased frequencies of classical monocytes and decreased frequencies of transitional and nonclassical monocytes when compared to the ASD^+^GI^−^ and TD groups.

Classical monocytes are the most abundant of the three subpopulations of monocytes, compared to the relatively smaller populations of transitional and nonclassical monocytes [24]. Classical monocytes are phagocytic cells that are primed to produce reactive oxygen species (ROS), secrete proinflammatory cytokines and increase migration [24]. Classical monocytes give rise to non-classical monocytes, of which the intermediate step is the transitional monocyte subset [34]. Transitional monocytes are similar to classical monocytes in their ability to initiate inflammatory processes, phagocytize materials, and present antigens [24]. Defining the heterogeneity of transitional monocytes remains difficult, as current studies indicate a large degree of variation that is donor- and disease-specific, as well as significant overlap in gene expression with both their classical and non-classical counterparts [35,36]. Nonclassical monocytes patrol blood vessels via increased surface expression of adhesins and more closely resemble resident tissue macrophages in their mediation of barrier integrity [25]. Circulating nonclassical monocytes were found to differentiate into macrophages with wound healing phenotypes in the large intestine in a Nr4a1-dependent manner, and deficiencies of this key transcription factor are associated with increased intestinal inflammation and delayed wound healing downstream [37]. Although less populous in vivo than classical monocytes, nonclassical monocytes differ from both classical and transitional monocytes in that they are longer lived (7 days vs. only 1–2 days) in circulation, are integral to wound healing and tissue repair, and aid in maintenance of barrier functions, such as in the intestinal mucosa [37,38].

Relative to classical monocytes, nonclassical monocytes uniformly express low levels of CCR2 but high levels of HLA-DR, with the reverse being true for classical monocytes [1]. Statistical analysis of differences in CCR2^+^ nonclassical monocyte frequencies could not be accurately compared across the groups due to a lack of captured events likely attributed to the diminished CCR2 levels typical of this subtype of monocyte, especially in the ASD^+^GI^+^ group. This is not unexpected, as nonclassical monocytes have typically low cell surface CCR2 compared to other monocyte subtypes. Statistically, ASD^+^GI^−^ groups had higher frequencies of CCR2^+^ classical monocytes compared to the TD group, with the ASD^+^GI^+^ group differences compared to TD approaching but not reaching statistical significance, likely due to the relatively smaller sample size in the ASD^+^GI^+^ group [39]. In the ASD^+^GI^−^ group there were significantly fewer CCR2^+^ transitional monocytes compared to both the TD and ASD^+^GI^+^ groups. We also demonstrated that the ASD^+^GI^+^ group has altered nonclassical monocyte activation indicated by lower nonclassical monocyte HLA-DR expression. Furthermore, the ASD^+^GI^+^ group had consistently higher levels of classical monocytes expressing exhaustion markers PD-1, PD-L1, and combined PD-1 PD-L1 compared to the ASD^+^GI^−^ and TD groups.

Monocytes are a heterogeneous population of immature mononuclear phagocytes that can be recruited to multiple tissue sites through finely orchestrated extracellular and intracellular signals that cue reparative or inflammatory phenotypes [19]. However, chronic stimulation and persistent activation of circulating monocytes propagate and progress many debilitating inflammatory conditions [20]. Functionally, monocytes play diverse roles throughout the body and are involved in tissue development and homeostasis, host defense, the initiation and resolution of inflammation, as well as tissue repair [19]. Classical monocytes have many of the commonly recognized functions typically associated with monocytes. These functions include the production of inflammatory cytokines, phagocytosis of pathogens and debris, and the ability to migrate to tissues for further differentiation into monocyte-derived tissue macrophages [40]. Although they are a precursor to other monocyte subsets, classical monocytes may have alternate fates via differentiation to macrophages or dendritic cells [34]. These specific fates are known to depend on the local exogenous and endogenous signals in the tissue environments in which they reside, or alternatively maintain their identity following egress from tissues. In this study, children with ASD, regardless of GI status, have higher frequencies of classical monocytes and lower levels of nonclassical monocytes than their TD counterparts. This may be explained by their higher expression of CCR2 on average, as CCR2 aids in the mobilization of monocytes from the bone marrow into circulation [41]. Complementary to our findings of elevated CCR2 expression in ASD groups, previous data has shown increased levels of the CCR2 ligand MCP-1 in ASD groups [33]. However, it is also possible that there are increases in progenitor cells or monopoieses that alter classical monocyte frequencies in ASD. Since CCR2 is also required for monocyte diapedesis into tissues and given that monocytes can gradually supplement tissue macrophage populations, an increase in classical monocytes could translate to increases in tissue macrophages. This observation still requires further investigation, but in the context of GI issues, increased monocyte infiltration into the intestinal tissue has been shown in ASD. As anticipated, we found diminished levels of CCR2^+^ nonclassical monocytes in our groups. Furthermore, the population of nonclassical cells in the ASD^+^GI^+^ group is smaller than that of the ASD^+^GI^−^ and TD groups. Interestingly, there may be a direct connection between the scarcity of nonclassical monocytes, known to facilitate wound healing and barrier integrity at the intestinal mucosa, in the ASD^+^GI^+^ group and the exacerbation of their GI symptoms and pathologies [37]. Nonclassical monocytes are reportedly involved in patrolling and maintaining endothelial and epithelial barriers, such as the gut epithelia [37]. In this role, non-classical monocytes mediate tissue repair via removing dying cells and promoting an “anti-inflammatory” environment. The significantly diminished frequency of nonclassical monocytes in the ASD^+^GI^+^ population could partially explain why children with ASD with GI issues have intestinal barrier integrity issues [15]. Given their roles in patrolling endothelial barriers, this finding may have implications for GI barrier integrity and potentially the blood-brain barrier, as these barrier sites are disrupted in ASD and also influenced by the nonclassical monocyte population [25].

The activation of monocytes induces the production of inflammatory cytokines, upregulation of costimulatory molecules that can induce adaptive immune responses, and engulfment of debris, dying cells, and/or pathogens [34]. Monocyte activity is tightly regulated to inhibit excessive inflammation and subsequent tissue damage while remaining active enough to ensure the elimination of potential infectious or noxious threats. The PD-1/PD-L1 system can help regulate monocyte activation, and dysregulation of this system is associated with higher risks of infection and poor outcomes in children and adults with sepsis [42,43]. In this study, the ASD^+^GI^+^ group had consistently higher levels of PD-1, PD-L1, and combined PD-1 PD-L1 co-expressing classical monocytes compared to the ASD^+^GI^−^ and TD groups. These findings of altered activation markers may explain recent findings from our group, revealing that ASD children have monocytes that cannot regulate their activation at the transcriptional level [22]. Poor control of monocyte activation may pose young children at risk for developing severe infections, an established environmental variable associated with ASD development [44].

Previous data from our laboratory has demonstrated that T cell subset frequency and distribution differ in children with ASD and comorbid GI issues, though the role of innate immune cells in the exacerbation of these symptoms has not been as thoroughly investigated [9]. Utilizing current knowledge of the frequency and roles of monocytes and monocyte-derived immune cells (i.e., macrophages) in the exacerbation of gastrointestinal diseases may allow us to contextualize the findings of our study and better understand the link between ASD and GI issues. “Monocytosis” is defined as a significant increase in blood-circulating monocytes, which we found to be the case in classical monocyte populations of the ASD^+^GI^+^ groups compared to the non-GI groups [45]. Other groups have demonstrated that patients with IBD and monocytosis exhibited worsened disease outcomes and increased likelihood for hospitalization compared to IBD patients without monocytosis [46]. Shifting our focus to nonclassical monocytes, previous studies have found a correlation between the depletion of this subtype and delayed wound healing in the gut [37]. Similarly, our study found greatly reduced non-classical monocytes in both ASD groups compared to the TD group, with statistical significance noted between the ASD^+^GI^+^ and TD groups. Still, the underlying biological cause for reduced nonclassical monocyte levels has yet to be fully understood. Some research has indicated that the maturation of intestinal macrophages is arrested at the immature developmental stage during IBD and is associated with the deficiency of monocyte chemotactic protein-induced protein 1 (MCPIP-1) [46]. Future investigations into the presence or absence of key markers along the monocyte-macrophage lineage in children with ASD and GI issues may support our current findings.

There are a few limitations within our preliminary study that we would like to address. We acknowledge the inability to stratify our data according to sex, which is an important variable to consider in ASD, and thus we intend to focus our efforts on recruiting females. We also recognize the small sample size within our groups. GI symptoms relied on validated assessments but were still based on parental reports and not clinician evaluations. We had no data on diet or potential microbiota changes in each group. Lastly, the inclusion of a TD group with GI symptoms would have allowed us to further characterize the uniqueness of our ASD^+^GI^+^ group; therefore, our recruitment efforts will focus on obtaining a well-powered fourth group.

## 5. Conclusions

The evaluation of monocyte populations and their surface markers has not been documented in ASD in the context of the presence of GI issues. We sought to understand how the monocyte populations and their expression of surface markers change with respect to ASD and the presence of GI symptoms. We were able to find changes in the frequencies of classical, transitional, and nonclassical monocyte populations that varied according to diagnosis and presence of GI symptoms. There is also evidence for altered classical monocyte activation in our ASD^+^GI^+^ group, as they uniquely had higher CCR2 expression and higher exhaustion marker PD-1 and PD-L1 expression. Our results provide more insight as to how the presence of comorbidities influences the immune cell landscape in ASD.

## Figures and Tables

**Table 1:** Demographic information for CHARGE participants.

Demographic Info.	TD	ASD^+^GI^−^	ASD^+^GI^+^
**# of subjects**	9	17	8
**Age range (years)**	5.25–9.25	5.25–9.92	4.17–9.83
**Sex (male/female)**	7/2	11/5 (1 unknown)	7/1
**ABC total score (median)**	6	34	29
**GIH total score (median)**	0	2	5

**Table 2:** Flow cytometry panel. All antibodies were purchased from BioLegend.

Antibody	Fluorochrome	Catalog #	Lot #
CD14	BV421	325628	B286545
CD16	APC	302012	B300870
CCR2	Alexa Fluor 488	357226	B279222
HLA-DR	APC/Fire 750	307658	B283532
PD-1	PE	329906	B252643
PD-L1	PerCP-Cy5.5	329738	B286901

**Table 3:** Mean Differences of activation markers in monocyte populations.

Marker	Monocyte Subpopulation	Group	Mean ± SEM (%)
		TD	43.30 ± 14.81
HLA-DR^+^	Classical (CD14^+^CD16^−^)	ASD^+^GI^−^	48.84 ± 11.22
		ASD^+^GI^+^	37.23 ± 13.82
		TD	32.48 ± 11.89
HLA-DR^+^	Transitional (CD14^+^CD16^+^)	ASD^+^GI^−^	24.43 ± 8.26
		ASD^+^GI^+^	40.16 ± 11.25
		TD	34.20 ± 13.00
HLA-DR^+^	Nonclassical (CD14^lo^CD16^+^)	ASD^+^GI^−^	52.60 ± 8.92
		ASD^+^GI^+^	24.36 ± 10.70
		TD	50.99 ± 13.88
HLA-DR^−^	Classical (CD14^+^CD16^−^)	ASD^+^GI^−^	62.59 ± 8.24
		ASD^+^GI^+^	85.60 ± 4.19
		TD	10.71 ± 5.29
HLA-DR^−^	Transitional (CD14^+^CD16^+^)	ASD^+^GI^−^	3.68 ± 1.26
		ASD^+^GI^+^	11.00 ± 5.28
		TD	26.16 ± 9.62
PD-1^+^	Transitional (CD14^+^CD16^+^)	ASD^+^GI^−^	18.76 ± 6.82
		ASD^+^GI^+^	0.19 ± 0.13
		TD	39.45 ± 14.58
PD-L1^+^	Transitional (CD14^+^CD16^+^)	ASD^+^GI^−^	26.21 ± 8.01
		ASD^+^GI^+^	17.09 ± 5.67
		TD	46.40 ± 13.81
PD-1^+^PD-L1^+^	Transitional (CD14^+^CD16^+^)	ASD^+^GI^−^	33.88 ± 10.90
		ASD^+^GI^+^	40.54 ± 13.07

## Data Availability

The original contributions presented in this study are included in the article. Further inquiries can be directed to the corresponding author(s).
